# The effect of latent *Mycobacterium tuberculosis* infection on ovarian reserve and pregnancy outcomes among infertile women in China

**DOI:** 10.1186/s12879-025-12424-x

**Published:** 2025-12-23

**Authors:** Xiaobing Yang, Haitian Chen, Xiangyi Kong, Taisheng Lai, Hailong Liang, Zhihao Chen, Tonghua Wu, Meilan Mo, Yuye Li

**Affiliations:** 1 Shenzhen Key Laboratory of Reproductive Immunology for Peri- implantation, Shenzhen Zhongshan Institute for Reproductive Medicine and Genetics, Shenzhen Zhongshan Obstetrics & Gynecology Hospital, Shenzhen, Guangdong China; 2 Guangdong Engineering Technology Research Center of Reproductive Immunology for Peri-implantation, Shenzhen, Guangdong China; 3 No. 1001, Fuqiang Road, Futian District, Shenzhen, Guangdong 518045 China

**Keywords:** Latent *Mycobacterium tuberculosis* infection, Ovarian reserve, Pregnancy outcome, Assisted reproductive technology

## Abstract

**Background:**

Latent *Mycobacterium tuberculosis* infection (LTBI) is common among patients with infertility, yet its effects on ovarian reserve function and pregnancy outcomes in women undergoing assisted reproductive technology (ART) remain unclear. This study aimed to investigate the impact of LTBI on ovarian reserve and clinical pregnancy outcomes in infertile women treated with ART.

**Methods:**

A total of 429 infertile female patients undergoing ART were enrolled in this study. Based on QuantiFERON-TB Gold Plus (QFT-Plus) results, patients were divided into LTBI and non-LTBI groups. Ovarian reserve markers and pregnancy outcomes were compared between the two groups.

**Results:**

Women in the LTBI group exhibited significantly lower anti-Müllerian hormone (AMH) levels than those in the non-LTBI group (3.42 ± 2.20 vs. 4.53 ± 3.30, *p* < 0.05). Although the LTBI group showed a lower clinical pregnancy rate (57.14% vs. 68.58%, *p* = 0.075), as well as reduced biochemical pregnancy rates (69.84% vs. 75.41%) and an increased rate of ectopic pregnancy (5.26% vs. 1.19%), these differences did not reach statistical significance (*p* > 0.05).

**Conclusions:**

The findings indicate that LTBI in women undergoing ART is associated with a significantly diminished ovarian reserve, as evidenced by lower AMH levels, and may potentially lead to adverse pregnancy outcomes, although these outcomes did not differ significantly between the groups.

## Background

Infertility is defined as the inability to conceive after 12 months of regular, unprotected intercourse [1]. As a major global health challenge, it is estimated that approximately 15% of reproductive-age couples experience infertility. This trend is particularly evident in China, where the age-standardized rates (ASR) of infertility have shown an upward trend, rising from 2,944.84 to 3,135.64 per 100,000 population between 1990 and 2021, reflecting a 6.5% increase over this three-decade period [2]. With advancements in modern medical technology, assisted reproductive technology (ART), ​such as in vitro fertilization (IVF), has become a primary and effective treatment for female infertility. Common causes of infertility include ovulatory dysfunction, diminished ovarian reserve, uterine factors, tubal disease, and male factors [1]. In recent years, there has been increasing recognition of the role of chronic infections in reproductive health. Consequently, growing evidence suggests that certain infectious diseases, particularly tuberculosis (TB), may also play a significant role in female infertility [3].

TB, caused by *Mycobacterium tuberculosis* (*M. tuberculosis)*, is a major global public health issue. According to the World Health Organization (WHO), China is one of the eight countries that account for over two-thirds of global TB cases, with China itself representing 6.8% of the total burden in 2023 [4]. While active TB directly contributes to infertility through direct organ damage, emerging evidence suggests that latent *Mycobacterium tuberculosis* infection (LTBI) may also impair reproductive function through mechanisms that remain poorly understood [5]. LTBI is defined as a state of persistent immune response to *M. tuberculosis* antigenic stimulation without clinical manifestations of active TB or imaging changes [6, 7]. At present, the Tuberculin Skin Test (TST) and Interferon-Gamma Release Assay (IGRA) are the primary immunological tools for the detection of LTBI. These tests measure the cell-mediated immune response to *M. tuberculosis*-specific antigens and are widely used in clinical and public health settings [8]. The high global burden of LTBI underscores its public health importance. Systematic reviews indicate that nearly a quarter of the global population is infected, with prevalences of 24.8% (IGRA-based) and 21.2% (TST-based) [9]. This pattern is similarly observed in China, where studies report an LTBI rate of 20.34% among adults aged ≥ 15 years, a prevalence that increases with age [10]. These findings highlight LTBI as a widespread condition that could significantly impact the fertility population.

*M. tuberculosis* can affect not only the lungs but also the lymph nodes, bones, kidneys, and reproductive organs, causing extrapulmonary TB [11]. Notably, the genital tract is a recognized site for extrapulmonary TB, often resulting from the hematogenous spread of the bacterium from a primary infection focus. Consequently, genital tuberculosis (GTB) is a major cause of female infertility, particularly in high-TB-burden regions [12]. Anti-Müllerian Hormone (AMH) and Antral Follicle Count (AFC) are sensitive indicators for ovarian reserve function [13]. AMH, secreted by ovarian granulosa cells, reflects the size of the primordial follicle pool, while AFC provides a direct ultrasonographic measure of the growing follicle cohort, collectively representing a woman’s ovarian reserve and potential fertility.​ Studies have shown that infertile women with latent genital tuberculosis (LGTB) have significantly lower AMH and AFC levels than the uninfected group, revealing that LGTB infection may impair ovarian reserve function [14, 15]. Therefore, the inclusion of TB infection status, whether active or latent, is essential when screening for causes of infertility in women.

As ART has become a cornerstone of infertility treatment, understanding the potential impact of LTBI on ovarian function and ART outcomes is crucial. While most studies have focused on active TB, the role of LTBI—particularly in high-burden regions such as China—has been largely overlooked. To address this gap, we aimed to comprehensively evaluate the impact of LTBI on both ovarian reserve and pregnancy outcomes in women undergoing ART. This study provides crucial evidence to inform screening strategies and clinical management for this patient population.

## Methods

### Study design

This was a retrospective cohort study of infertile women who underwent ART from April to June 2023 at the Reproductive Center of Shenzhen Zhongshan Obstetrics & Gynecology Hospital, China.

### Study population

**Fig. 1:**
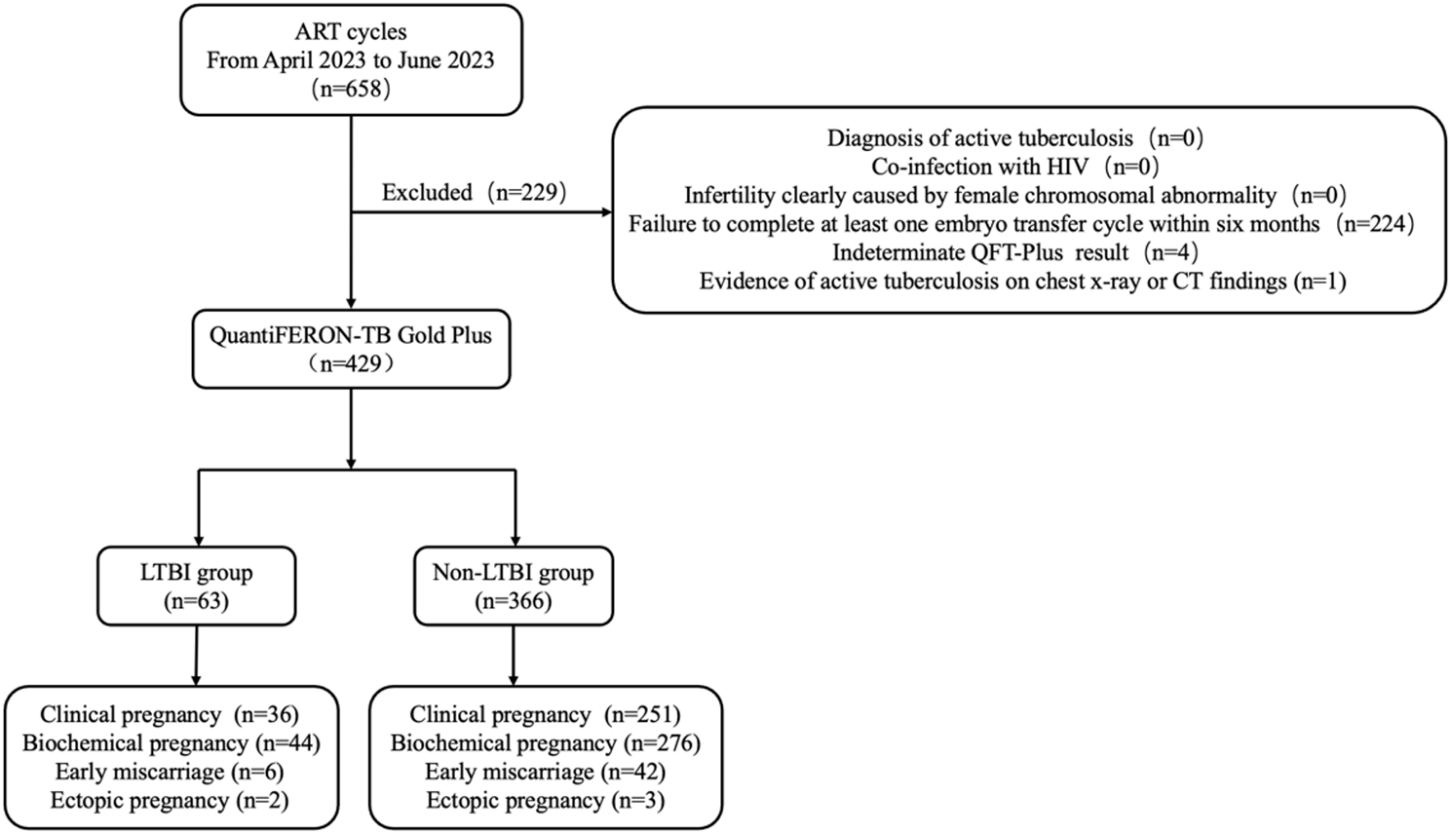
Flow chart of the selection of cases for inclusion in this study. A total of 658 infertile patients who visited the fertility center from April to June 2023 and underwent QuantiFERON-TB Gold Plus were initially enrolled in this study. Of these, 229 participants were excluded based on predefined exclusion criteria. Clinical statistics were performed on the remaining 429 patients

The study population was selected based on the following criteria. Inclusion criteria were: (1) female aged between 20 and 43 years. (2) routine indications for ART. (3) completion of an IGRA prior to ART. 4༉no history of anti-tuberculosis treatment (ATT). Exclusion criteria were: (1) diagnosis of active tuberculosis. (2) HIV co-infection. (3) infertility due to known female chromosomal abnormalities. (4) failure to undergo a complete cycle of in vitro fertilization-embryo transfer (IVF-ET) or intracytoplasmic sperm injection-embryo transfer (ICSI-ET) within six months after IGRA testing. (5) an indeterminate QuantiFERON-TB Gold Plus (QFT-Plus) result. (6) radiological evidence of active tuberculosis on chest X-ray or computed tomography (CT) scan. These data were ​routinely recorded in the electronic medical records​ as part of the standard infertility workup and ​were retrospectively reviewed and extracted by the research team​ for analysis. The recruitment flowchart is presented in Fig. 1.

All women underwent IGRA testing within six months prior to ART. In cases of a positive IGRA test, a chest X-ray or CT was performed to determine whether the woman had active TB. Each woman underwent endoluminal color Doppler ultrasound (transvaginal ultrasound, TVS) on days 2–4 of the menstrual cycle, during which follicles ranging from 2 to 9 mm in diameter were counted in both ovaries to determine the AFC. Blood samples were also collected for the measurement of AMH levels and basal sex hormone tests, including follicle stimulating hormone (FSH), luteinizing hormone (LH), estradiol (E2), and progesterone (PG), to assess ovarian reserve and basal ovarian function. Following controlled ovarian stimulation, the number of oocytes retrieved during the ovum pick-up procedure was recorded as the final indicator of ovarian response..

### Grouping of study subjects

Based on the peripheral blood QFT-Plus test before ART treatment, subjects were categorized into two groups: the LTBI group, comprising those with a positive QFT-Plus test, and the non-LTBI group, consisting of those with a negative QFT-Plus test.

### QuantiFERON-TB gold plus assay

The QFT-Plus assay (QIAGEN GmbH, Germany) was performed on peripheral blood samples collected from all enrolled patients, according to the manufacturer’s instructions. In brief, whole blood was collected into lithium heparin tubes and subsequently aliquoted into QFT-Plus tubes containing *M. tuberculosis*-specific antigens (TB1 and TB2). Following a 16- to 24-hour incubation at 37 °C, the plasma supernatant was harvested by centrifugation. The concentration of interferon-gamma (IFN-γ) in the plasma in response to the antigens was then quantified using an enzyme-linked immunosorbent assay (ELISA). Results were interpreted as positive, negative, or indeterminate based on the manufacturer’s recommended criteria, which account for the nil-, antigen-, and mitogen-tube IFN-γ values.

### Blood biochemical examination

Peripheral blood was collected from all participants on days 3–5 of a natural menstrual cycle. Following centrifugation, serum levels of baseline sex hormones (FSH, LH, E2, and PG) and AMH were measured using chemiluminescence immunoassays, performed in accordance with the manufacturers’ protocols (Roche Diagnostics, Basel, Switzerland for sex hormones; YHLO Biotech, Shenzhen, China for AMH).

### AFC measurement

The AFC numbers were counted using a Color Doppler ultrasound system (model LOGIQ-400BW, GE Healthcare, Milwaukee, WI, USA) on day 2 of the menstrual cycle. ​The examination was performed following a standardized departmental protocol to ensure measurement consistency.

### Pregnancy outcome

Pregnancy outcomes were defined as follows [5]:

- Clinical pregnancy (primary outcome): Defined as the presence of a gestational sac within the uterus, with or without a detectable fetal heartbeat, confirmed by transvaginal ultrasound 4–6 weeks after embryo transfer.
- Biochemical pregnancy: Defined as a serum human chorionic gonadotropin (hCG) level exceeding 10 U/L, measured 14 days after embryo transfer.
- Early miscarriage rate: Defined as an ultrasound-confirmed spontaneous pregnancy loss occurring before 12 weeks of gestation.
- Ectopic pregnancy rate: Defined as a pregnancy diagnosed as being implanted outside the uterine cavity by transvaginal ultrasound and/or surgical visualization.

### Outcome calculation formulas

Clinical pregnancy rate (%) = (Number of patients with a gestational sac detected by ultrasound at 4–6 weeks after embryo transfer / Total number of embryo transfer cycles) × 100%.

Biochemical pregnancy rate (%) = (Number of patients with serum hCG > 10 U/L at 14 days after embryo transfer / Total number of embryo transfer cycles) × 100%.

Early miscarriage rate (%) = (Number of spontaneous miscarriages within the first 12 weeks of gestation / Total number of clinical pregnancy cycles) × 100%.

Ectopic pregnancy rate (%) = (Number of ectopic pregnancies / Total number of clinical pregnancy cycles) × 100%.

### Statistical analysis

Statistical analysis was performed using SPSSAU (SPSSAU Inc., Guangzhou, China). ​The normality of the distribution for continuous variables was assessed using the Shapiro-Wilk test. Data that followed a normal distribution are presented as mean ± standard deviation (SD), and comparisons between two groups were made using the independent samples t-test. Non-normally distributed data are expressed as median with interquartile range [M (Q1, Q3)], and the Mann-Whitney U test was used for group comparisons. Categorical data are expressed as percentages (%), and group differences were analyzed using the Chi-square test or Fisher’s exact test, as appropriate. Linear regression was used to assess the association between LTBI status and AMH levels. Multiple logistic regression was employed to evaluate the influence of LTBI status on pregnancy outcomes. A two-tailed *p*-value of < 0.05 was considered statistically significant.

## Results

### Comparison of baseline data between LTBI and non-LTBI groups

A total of 658 infertile women were initially screened for eligibility. Of these, 229 were excluded, with the main reasons being an indeterminate QFT-Plus result, or failure to complete an ART cycle, as detailed in Fig. 1. Consequently, the final study population comprised 429 patients, who were stratified into a LTBI group (*n* = 63) and a non-LTBI group (*n* = 366).

The baseline characteristics of the two groups are summarized in Table 1. A significant difference was observed in body mass index (BMI), with the LTBI group having a higher mean value than the non-LTBI group (23.18 ± 3.66 kg/m² vs. 21.97 ± 3.21 kg/m², *p* = 0.008). However, the groups were comparable with regard to other demographic, clinical, and embryological parameters, including age, duration of infertility, baseline hormone levels, and assisted reproduction procedural details.

**Table 1:** Comparison of basic and clinical information for the LTBI, non-LTBI groups

Characteristics	LTBI group (*n* = 63)	non-LTBI group (*n* = 366)	χ^2^/Z/t Value	*p*-Value
**Female age at cycle start**,** years (mean ± SD)**	33.08 ± 5.06	32.79 ± 4.24	-0.487	0.627
**Duration of infertility**,** years (mean ± SD)**	3.30 ± 2.60	3.30 ± 2.57	0.005	0.996
**BMI**,** kg/m**^**2**^ **(mean ± SD)**	23.18 ± 3.66	21.97 ± 3.21	-2.685	0.008*
**Baseline hormone (mean ± SD)**				
FSH, mIU/mL	5.91 ± 3.38	6.18 ± 4.65	0.445	0.656
LH, mIU/mL	5.96 ± 3.78	6.48 ± 6.64	0.606	0.545
E2, pg/mL	145.35 ± 298.13	102.90 ± 130.64	-1.112	0.270
PG, ng/mL	2.11 ± 4.05	2.69 ± 4.81	0.996	0.322
**Type of infertility (%)**			0.087	0.768
Primary infertility	55.56 (35/63)	53.55 (196/366)		
Secondary infertility	44.44 (28/63)	46.45 (170/366)		
**Cause of infertility (%)**			8.039	0.154
Pelvic and tubal factors	36.51 (23/63)	28.93 (105/363)		
Ovulatory disorders	3.17 (2/63)	13.22 (48/363)		
Endometriosis	4.76 (3/63)	9.92 (36/363)		
Unexplained	22.22 (14/63)	20.11 (73/363)		
Male factors	19.05 (12/63)	17.08 (62/363)		
Combined factors	14.29 (9/63)	10.74 (39/363)		
**Cycles with different technologies (%)**			2.324	0.313
IVF	77.78 (49/63)	79.78 (292/366)		
ICSI	20.63 (13/63)	15.30 (56/366)		
Others	1.59 (1/63)	4.92 (18/366)		
**No. of embryos transferred**	80	472		
**Embryo type (%)**			0.192	0.661
Cleavage	20.63 (13/63)	18.31 (67/366)		
Blastocyst	79.37 (50/63)	81.69 (299/366)		
**Embryo transfer (%)**			1.505	0.471
Fresh	22.22 (14/63)	19.13 (70/366)		
Frozen	74.60 (47/63)	79.51 (291/366)		
Fresh and frozen	3.17 (2/63)	1.37 (5/366)		

Note. LTBI: latent *Mycobacterium tuberculosis* infection; non-LTBI: non-latent *Mycobacterium tuberculosis* infection; BMI: body mass index; FSH: follicle-stimulating hormone; LH: luteinizing hormone; E2: estradiol; PG: progesterone; IVF: in vitro fertilization; ICSI: intracytoplasmic sperm injection. *: *p* value < 0.05

### Assessment of ovarian reserve function in infertile patients in LTBI and non-LTBI groups

Ovarian reserve function was evaluated in infertile women using AMH and AFC. AMH levels were significantly lower in the LTBI group than in the non-LTBI group (3.42 ± 2.20 ng/mL vs. 4.53 ± 3.30 ng/mL, *p* = 0.001). Although not statistically significant, both the AFC and the number of oocytes retrieved showed consistent decreasing trends in the LTBI group (AFC: 14.47 ± 7.19 vs. 16.03 ± 9.04, *p* = 0.196; number of oocytes retrieved: 13.08 ± 7.06 vs. 15.06 ± 8.04, *p* = 0.067). (Table 2).

**Table 2:** Ovarian reserve parameters in the LTBI and non-LTBI groups

Characteristics	LTBI group (*n* = 63)	non-LTBI group (*n* = 366)	Z/t Value	*p*-Value
AMH (ng/mL)	3.42 ± 2.20	4.53 ± 3.30	3.406	0.001*
AFC	14.47 ± 7.19	16.03 ± 9.04	1.296	0.196
Number of oocytes retrieved​	13.08 ± 7.06	15.06 ± 8.04	1.837	0.067

Note. LTBI: latent *Mycobacterium tuberculosis* infection; non-LTBI: non-latent *Mycobacterium tuberculosis* infection; AMH: anti-Müllerian hormone; AFC: antral follicular count

*: *p* value < 0.05

The association between LTBI status and AMH levels was further assessed using linear regression. In an unadjusted model, LTBI status was a significant negative predictor of AMH (β = -1.112, 95% CI: − 1.960 to − 0.263, *p* = 0.010). After adjustment for age, the association remained significant (adjusted β = − 1.062, 95% CI: − 1.886 to − 0.239, *p* = 0.012). The results of the regression analysis are presented in Table 3..

**Table 3:** Linear regression analysis of factors associated with AMH levels

Variables	Unadjusted			Adjusted			
	β	95%CI	*p*-Value	β	95%CI	*p*-Value	
**Latent Mycobacterium tuberculosis infection**							
non-LTBI	Ref	Ref	Ref	Ref	Ref	Ref	
LTBI	-1.112	-1.960 ~ -0.263	0.010*	-1.062	-1.886 ~ -0.239	0.012*	
**Female age at cycle start**	-0.181	-0.249 ~ -0.114	0.000*	-0.179	-0.246 ~ -0.112	0.000*	

Note. β: regression coefficient; LTBI: latent *Mycobacterium tuberculosis* infection; non-LTBI: non- latent *Mycobacterium tuberculosis* infection; Ref: Reference

*: *p* value < 0.05

### Pregnancy outcomes of infertile patients in LTBI and non-LTBI groups

Following the assessment of ovarian reserve, pregnancy outcomes were compared between the groups. Based on the primary pregnancy outcome results, the clinical pregnancy rate in the LTBI group was only 57.14%, which ​was lower than that of the non-LTBI group at 68.58%, although the difference did not reach statistical significance (*p* = 0.075). Regarding secondary outcomes, the LTBI group had lower biochemical pregnancy rates (69.84% vs. 75.41%), lower early miscarriage rates (16.67% vs. 16.73%), and higher ectopic pregnancy rates (5.26% vs. 1.19%). However, no statistically significant differences were found for these outcomes (*p* > 0.05, Table 4), as assessed using the standardized procedures detailed in the Statistical Analysis section.

**Table 4:** Pregnancy outcomes in the LTBI and non-LTBI groups

Pregnancy outcomes	LTBI group (*n* = 63)	non-LTBI group (*n* = 366)	χ^2^ Value	*p*-Value
**Primary outcome (%)**				
Clinical pregnancy rate	57.14 (36/63)	68.58 (251/366)	3.175	0.075
**Secondary outcome (%)**				
Biochemical pregnancy rate	69.84 (44/63)	75.41 (276/366)	0.879	0.348
Early miscarriage rate	16.67 (6/36)	16.73 (42/251)	0.000	0.992
Ectopic pregnancy rate	5.26 (2/38)	1.19 (3/253)	0.216	0.129

Note. LTBI: latent *Mycobacterium tuberculosis* infection; non-LTBI: non-latent *Mycobacterium tuberculosis* infection; IVF: in vitro fertilization; ICSI: intracytoplasmic sperm injection

*: *p* value < 0.05

In the multiple logistic regression model adjusted for body mass index, female age, infertility type, number of embryos transferred, and embryo type, LTBI status was not significantly associated with clinical pregnancy (adjusted OR = 0.577, 95% CI: 0.319 to 1.041, *p* = 0.068). This indicates a non-significant trend toward reduced pregnancy rates associated with LTBI. The full regression analysis results are presented in Table 5.

**Table 5:** Logistic regression analysis of the effect of potential predictor variables on clinical pregnancy outcomes

Variables	Unadjusted			Adjusted			
	OR	95%CI	*P*-Value	OR	95%CI	*p*-Value	
**Latent Mycobacterium tuberculosis infection**							
non-LTBI	Ref	Ref	Ref	Ref	Ref	Ref	
LTBI	0.611	0.354 ~ 1.054	0.077	0.577	0.319 ~ 1.041	0.068	
**Female age at cycle start**	0.922	0.879 ~ 0.966	0.001*	0.973	0.923 ~ 1.026	0.314	
**BMI**	0.999	0.940 ~ 1.062	0.982	1.032	0.964 ~ 1.105	0.365	
**Type of infertility**							
Primary infertility	Ref	Ref	Ref	Ref	Ref	Ref	
Secondary infertility	0.455	0.302 ~ 0.685	0.000*	0.529	0.340 ~ 0.821	0.004*	
**No. of embryos transferred**	0.516	0.334 ~ 0.796	0.003*	1.561	0.787 ~ 3.098	0.202	
**Embryo type**							
Cleavage	Ref	Ref	Ref	Ref	Ref	Ref	
Blastocyst	4.656	2.792 ~ 7.765	0.000*	5.485	2.533 ~ 11.879	0.000*	

Note. OR: odds ratio; CI: confidence interval; LTBI: latent *Mycobacterium tuberculosis* infection; non-LTBI: non- latent *Mycobacterium tuberculosis* infection; Ref: Reference; BMI: body mass index

*: *p* value < 0.05

## Discussion

In this study, we investigated the impact of LTBI on ovarian reserve function and pregnancy outcomes in women undergoing ART. Our findings suggest that LTBI may have a negative effect on ovarian reserve, as indicated by significantly lower AMH levels in LTBI-positive women compared to uninfected controls. Additionally, an overall decline in AFC was observed in the LTBI group, although the difference did not reach statistical significance. Regarding pregnancy outcomes, the clinical and biochemical pregnancy rates were lower in the LTBI group compared to the non-LTBI group. While these differences were not statistically significant, the observed trends indicate a potential adverse effect of LTBI on reproductive success. Our study extends the known impact of tuberculosis on fertility beyond the structural damage caused by active genital TB, suggesting that LTBI could be an underappreciated factor in ovarian function and reproductive success.

The prevalence of LTBI observed in our study exhibits marked variability when compared to findings from other studies. For instance, while a cohort from the United States reported a prevalence of 7.7% [16], a study on patients undergoing in vitro fertilization-frozen embryo transfer (IVF-FT) reported 35.4% [17]. These discrepancies likely stem from differences in geographical location, study population characteristics, and methodologies.

AMH and AFC are two important indicators for assessing ovarian reserve function, and they are often correlated but do not always correspond exactly [18]. AMH, secreted by granulosa cells, primarily reflects the quantity of primordial follicles and small antral follicles within the ovary, while AFC specifically measures the number of antral follicles. These two biomarkers offer distinct but complementary insights into ovarian reserve. A clinical study involving 884 infertile women found that those with LGTB showed a significant reduction in both AMH and AFC levels, with a 15% decrease in both markers, and a more pronounced decline in ovarian reserve after excluding Polycystic Ovary Syndrome (PCOS) patients [15]. Building on the established utility of these biomarkers, the present study applied AMH and AFC to evaluate ovarian reserve in LTBI patients. In this study, ovarian reserve function indicators (AMH and AFC) were lower in the LTBI group than in the non-LTBI group. Specifically, AMH levels showed a statistically significant difference, whereas AFC did not show a significant change, despite a downward trend. This pattern of diminished ovarian reserve was further reflected in a reduced number of oocytes retrieved in the LTBI group, suggesting a corresponding decrease in the ovary’s response to stimulation. Latent genital tuberculosis infection (LGTBI) represents a reproductive system-specific manifestation of LTBI, which is more likely to cause clinically detectable damage to the reproductive system. This discrepancy could potentially explain the variations observed between the two studies. Our study suggests LTBI initially impairs granulosa cell function, disrupts follicular development, and ultimately alters AMH and AFC levels. The lower AMH levels in the LTBI group raise important questions about the potential long-term effects of LTBI on fertility. Early detection and treatment of LTBI could help mitigate these effects and preserve ovarian function.

Recent metabolomics studies on LTBI have revealed significant changes in glycerophospholipid, steroid hormone, and bile acid metabolism, which could provide insights into how LTBI affects ovarian reserve [19]. These metabolic disorders may impair ovarian function through several key pathways: Lysophosphatidylcholines (lysoPCs), key components of glycerophospholipid metabolism, activate extracellular signal-regulated kinase (ERK) and protein kinase C (PKC), both of which are crucial for follicular development and oocyte maturation [20, 21]. Cortisol promotes follicular cell survival via AMH signaling, protecting oogenesis [22]. In animal models of cholestatic bile duct ligation (BDL), bile acids have been shown to impair mitochondrial function and induce oxidative stress, ultimately compromising ovarian function [23].

Furthermore, inflammatory damage represents another critical mechanism. A study by Datta et al. reported significantly elevated levels of the pro-inflammatory cytokines IFN-γ, interleukin-6 (IL-6) and tumor necrosis factor-alpha (TNF-α) in patients with LGTBI, along with several other inflammatory factors [24]. Li et al. reported that increased intrafollicular inflammation may be associated with diminished ovarian reserve (DOR), and high levels of IL-6 in follicles may negatively affect embryo quality [25]. Collectively, these findings highlight potential mechanisms by which LTBI could impair ovarian reserve, supporting the hypothesis that LTBI contributes to ovarian dysfunction through both metabolic disruption​ and ​inflammatory damage.

Female genital tuberculosis (FGTB), as an advanced stage of tuberculous infection, provides a comparative model for understanding the potential impact of LTBI on fertility. While FGTB directly damages reproductive structures like the fallopian tubes and endometrium, leading to infertility [26], its pathogenesis also involves immune dysregulation characterized by a Th1-dominant response and inflammatory pathway activation [27]. ​Crucially, these immunological mechanisms—such as altered cytokine secretion and inflammatory damage—are shared with LTBI, albeit at a subclinical level. This Th1-dominant immune milieu can impair endometrial receptivity, interfere with embryo implantation [28], and increase the risk of miscarriage, consistent with the bias observed in women with recurrent pregnancy loss [29, 30]. This highlights a continuum of tuberculous impact on reproduction.

A study comparing pregnancy outcomes in women with LTBI undergoing IVF-FET found significantly lower clinical pregnancy rate in the LTBI group, with LTBI identified as an independent risk factor for reduced pregnancy outcomes after adjusting for confounding variables [17]. Although our study did not find a statistically significant association, the observed trend of lower clinical pregnancy rates in the LTBI group aligns with the direction of this previously reported risk.​ This may be due to sample size limitations or variations in embryology techniques and embryo characteristics. Nonetheless, it is crucial to note that studies have shown that pregnancy outcomes in women with LGTB can be significantly improved following ATT [15]. Therefore, early detection and treatment of LTBI may potentially enhance pregnancy outcomes in infertile women.

Despite these insightful findings, our study has limitations that warrant consideration. Its retrospective, single-center design and small sample size may introduce selection bias and limit statistical power. Furthermore, the findings could be influenced by unmeasured confounders, including variations in ART protocols and embryology techniques, and their generalizability may be constrained by the short, 3-month observation period of this ongoing study. Therefore, our future research will aim to collect long-term, multi-center data with an expanded sample size and stricter inclusion criteria (e.g., confined age ranges and exclusion of conditions such as endometriosis) to validate and extend the current conclusions.

## Conclusion

Ovarian reserve function is significantly reduced in women with LTBI undergoing ART, and pregnancy outcomes may be adversely affected, even though statistical significance was not reached, observed trends warrant further investigation. These findings highlight the potential clinical value of early LTBI screening in infertility assessments. Future research should focus on confirming these associations and evaluating the benefit of preventive anti-tuberculosis therapy to optimize ART outcomes.

## Data Availability

The datasets used and/or analyzed during the current study available from the corresponding author on reasonable request.

## Abbreviations

AFC: Antral follicle count
AMH: Anti-Müllerian hormone
ART: Assisted reproductive technology
ASR: Age-standardized rates
ATT: Anti-tuberculosis treatment
BDL: Bile duct ligation
BMI: Body mass index
CT: omputed tomography
DOR: Diminished ovarian reserve
E2: Estradiol
ERK: Extracellular signal-regulated kinase
FGTB: Female genital tuberculosis
FSH: Follicle-stimulating hormone
GTB: Genital tuberculosis
ICSI-ET: Intracytoplasmic sperm injection–embryo transfer
IFN-γ: Interferon-gamma
IGRA: Interferon-gamma release assay
IL-6: Interleukin-6
IUI: Intrauterine insemination
IVF-ET: In vitro fertilization–embryo transfer
IVF-FT: In vitro fertilization–frozen embryo transfer
LGTB: Latent genital tuberculosis
LGTBI: Latent genital tuberculosis infection
LH: Luteinizing hormone
LTBI: Latent Mycobacterium tuberculosis infection
lysoPCs: Lysophosphatidylcholines
M. tuberculosis: Mycobacterium tuberculosis
PCOS: Polycystic Ovary Syndrome
PG: Progesterone
PKC: Protein kinase C
QFT-Plus: QuantiFERON-TB Gold Plus
TB: Tuberculosis
TNF-α: Tumor necrosis factor-alpha
TST: Tuberculin skin test
TVS: Transvaginal ultrasound
WHO: World Health Organization
